# Changes in resident microbiota associated with mice susceptibility or resistance to the intestinal trematode *Echinostoma caproni*

**DOI:** 10.1017/S0031182022001366

**Published:** 2022-11

**Authors:** Maria Álvarez-Izquierdo, Emma Fiallos, Paola Cociancic, J. Guillermo Esteban, Carla Muñoz-Antoli, Rafael Toledo

**Affiliations:** Área de Parasitología, Departamento de Farmacia y Tecnología Farmacéutica y Parasitología, Facultad de Farmacia, Universitat de València, Avda. Vicent Andrés Estellés s/n, 46100 Burjassot, Valencia, Spain

**Keywords:** *Echinostoma caproni*, interleukin-25, intestinal helminths, microbiota, resistance, Verrucomicrobia

## Abstract

*Echinostoma caproni* (Trematoda: Echinostomatidae) is an intestinal trematode with no tissue phases in the definitive host that has been extensively used as an experimental model to study the factors that determine resistance against intestinal helminths. In *E. caproni* infections in mice, interleukin-25 (IL-25) plays a critical role and it is required for the resistance to infection. However, little is known on the factors that determine its production. Primary *E. caproni* infection in mice is characterized by the development of chronic infections and elevated worm recovery, in relation to a local Th1 response with elevated production of interferon-*γ*. However, partial resistance against secondary *E. caproni* infections in ICR (Institute of Cancer Research) mice is developed after the chemotherapeutic cure of a primary infection and the innately produced IL-25 after pharmacological treatment. In this paper, we analyse the potential role of intestinal microbiota in the production of IL-25, and the subsequent resistance to infection. For this purpose, we analysed the production of IL-25 under conditions of experimental dysbiosis and also the changes in the resident microbiota in primary infections, pharmacological curation and secondary infections. The results obtained showed that resident microbiota play a major role in the production of IL-25 and the appearance of members of the phylum Verrucomicrobia as a consequence of the curation of the primary infection could be related to the partial resistance to secondary infection.

## Graphical abstract

**Figure:**
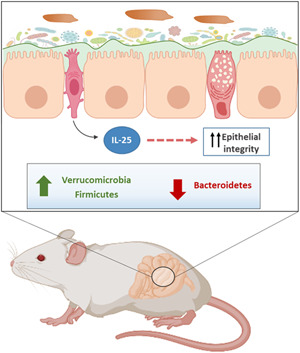

## Introduction

Intestinal helminth infections are among the most prevalent infections, both in humans and animals. In humans, it is estimated that more than 1.5 billion people are infected with 1 or more species of intestinal helminths, especially in Asia, Africa and Latin-America, causing a significant impact on human health, mainly in children (Hotez *et al*., 2008; Weatherhead and Hotez, 2015). Moreover, intestinal helminths cause a large amount of economic losses due to diminishing productivity and also the cost of anthelminthic treatments (Roeber *et al*., 2013). Due to the unavailability of vaccines, the development of new control strategies is needed and, in this context, the use of experimental models has served to increase our knowledge on the factors governing the resistance to intestinal helminths.

Evidence from studies using different experimental models suggests that resistance to intestinal helminths relies on the activation of a Th2-type immunity involving the production of interleukin-4 (IL-4), IL-5, IL-9 and IL-13 (Cortés *et al*., 2017). In this context, IL-25 has been regarded as the main regulatory cytokine responsible for the development of the protective Th2-biased immune response. IL-25 participates in the maintenance of the intestinal homoeostasis, tissue adaptation to external damage and tissue repair (Borowczyk *et al*., 2021; Varyani *et al*., 2022). IL-25 is mainly produced by epithelial tuft cells because of the chemosensory receptors that these cells present (Borowczyk *et al*., 2021), linking with type 2 immunity by stimulating production of IL-13 by type 2 innate lymphoid cells which, in turn, induces signal transducer and activator of transcription 6-mediated intestinal alterations leading to resistance to infection (Fallon *et al*., 2006; Owyang *et al*., 2006; Zhao *et al*., 2010; Angkasekwinai *et al*., 2013, 2017; Cortés *et al*., 2017; Varyani *et al*., 2022). In fact, emerging evidence shows that IL-25 is required for resistance to intestinal helminth infections (Muñoz-Antoli *et al*., 2016*a*).

Our group showed that mice susceptibility to primary infection with the intestinal trematode *Echinostoma caproni* depends on the inability of mice to respond to IL-25 production. However, mice develop partial resistance to secondary infection in relation to the innate production of IL-25 as a consequence of the curation of the primary infection (Muñoz-Antoli *et al*., 2016*a*, 2016*b*). Furthermore, Álvarez-Izquierdo *et al*. (2020*a*) demonstrated that type 2 response to secondary *E. caproni* infection in mice was unable to develop resistance in the absence of IL-25, confirming the requirement of IL-25 for resistance and showing that the role of this cytokine is not limited to regulatory functions, but also has a critical effector role in the resistance. However, the factors that determine the upregulation of IL-25 are not known, but there are several lines of evidence suggesting that it can be driven by resident gut microbiota (Zaph *et al*., 2008; Sawa *et al*., 2011; Donaldson *et al*., 2015; Watanabe *et al*., 2017). IL-25-mediated intestinal immune regulation is impaired in mice in the absence of microbiota (Sawa *et al*., 2011; Donaldson *et al*., 2015; Watanabe *et al*., 2017). Expression of ileal IL-25 is reduced in germ-free mice compared to wild-type mice, but exposure to environmental microbes induced IL-25 overexpression (Sawa *et al*., 2011; Donaldson *et al*., 2015). Furthermore, antibiotic treatment of mice significantly decreased the expression of gut IL-25 (Zaph *et al*., 2008).

To investigate the potential role of resident microbiota in the production of IL-25 and the subsequent resistance to infection we have used the experimental model of *E. caproni* in mice. *Echinostoma caproni* (Trematoda: Echinostomatidae) is an intestinal trematode with no tissue phase in the definitive host (Fried and Huffman, 1996; Toledo *et al*., 2009). Although *E. caproni* is able to parasitize a wide range of laboratory rodent hosts, its compatibility differs considerably between rodent species. In highly compatible hosts, such as mice, primary infection becomes chronic, while pharmacological healing of the primary infection induces a sudden production of IL-25. Secondary infection in the presence of IL-25 results in partial resistance to the challenge infection (Muñoz-Antoli *et al*., 2016*a*, 2016*b*; Álvarez-Izquierdo *et al*., 2020*a*, 2020*b*). Herein, we analyse the changes in resident microbiota induced by primary *E. caproni* infection in mice, cure of the primary and secondary infections with the aim to gain further insight into the potential role of microbiota in the production of IL-25 and resistance to infection. This experimental model allows us to analyse 3 different situations: (1) susceptibility in primary infections associated with the lack of IL-25; (2) changes induced by healing of the primary infections and (3) resistance to secondary infection in an environment with elevated levels of IL-25.

## Materials and methods

### Parasites, hosts and experimental infections

The strain of *E. caproni* has been described previously (Fujino and Fried, 1993). Encysted metacercariae of *E. caproni* were removed from the kidney and pericardial cavity of experimentally infected *Biomphalaria glabrata* snails and used to infect male ICR (Institute of Cancer Research) mice of the same age and weighing 30–35 g by gastric gavage (50 metacercariae each). The positivity of the infection was determined at necropsy or detection of eggs in stools as described previously (Toledo *et al*., 2004).

### Primary and secondary experimental infections

A total of 15 mice were infected and randomly allocated to 3 groups (infected, treated and secondarily infected) of 5 mice each. The characteristics of the primary infections were studied in the animals in the primary infected group. These animals were maintained as infected without additional treatment until the end of the experiment at 10 weeks post-primary infection (wppi). At 4 wppi, animals in the other 2 groups were treated with a double dose of praziquantel (pzq) of 100 mg kg^−1^ (pzq-treated and secondarily infected groups), orally administered on alternate days as described previously (Muñoz-Antoli *et al*., 2016*a*, 2016*b*). All mice belonging to these 2 groups responded to the treatment and reverted to the negative state as determined by coprological examination. Two weeks after treatment, all mice in the secondarily infected group were challenged with 50 metacercariae of *E. caproni* following the same procedure used in primary infections. Additionally, 5 mice were maintained uninfected and used as naïve controls. At 4, 6 and 10 wppi, stools from each individual mouse were collected to investigate the resident microbiota. The influence of the pharmacological treatment over the studied parameters was ignored since 4 additional mice were left uninfected, treated with pzq as described above and analysed as in the other animals. All the animals were individually maintained in cages under conventional conditions with food and water *ad libitum*. All mice were sacrificed and necropsied at the end of the experiment at 10 wppi.

The effect of the infection, pzq-treatment and reinfection on IL-25 production and its potential relation to changes in microbiota composition was studied on 45 additional mice. These animals were randomly allocated into 3 groups as described above (infected, treated and secondarily infected) of 15 each and the procedures described above were followed for each group. At 2, 4, 5, 6 and 10 wppi, 3 animals from each group were sacrificed and necropsied to study the production of IL-25.

### Induction of intestinal dysbacteriosis and IL-25 production

To analyse the effect of resident microbiota on the production of IL-25 in response to secondary *E. caproni* infections, a total of 10 mice were primarily infected and treated with pzq at 4 wppi. In 5 of these animals, dysbacteriosis was induced using a cocktail of broad-spectrum antibiotics. Mice received drinking water containing ampicillin (Sigma, Darmstadt, Germany) (1.0 g L^−^), metronidazole (Guinama, Valencia, Spain) (1.0 g L^−^), neomycin (Sigma) (1.0 g L^−^) and vancomycin (Sigma) (0.5 g L^−^) for 2 weeks before secondary infection with 50 metacercariae of *E. caproni*. The remaining 5 mice were secondarily infected at 2 weeks post-pzq treatment without antibiotic treatment. All mice were necropsied at 10 wppi and expression of IL-25 was compared between both groups of mice. Additionally, 5 mice were used to evaluate the potential effect of antibiotic treatment in the expression of IL-25. These mice primarily infected and treated with pzq and antibiotics followed the same procedure but were not secondarily infected. No changes in IL-25 were observed in these control animals.

### Total RNA extraction from intestinal tissues and relative quantification analysis of IL-25 by quantitative polymerase chain reaction (qPCR)

Total RNA was extracted from full-thickness sections of ileum of necropsied mice. Total RNA was isolated using a Real Total ARN Spin Plus kit (Durviz) according to the manufacturer's instructions. The cDNA was synthesized using a High Capacity cDNA Reverse Transcription kit (Applied Biosystems, Foster City, CA, USA).

For qPCR, 40 ng total RNA was reverse transcribed to cDNA and added to 10 *μ*L TaqMan Universal PCR Master Mix, No AmpErase UNG (2×), 1 *μ*L of the specified TaqMan gene expression assay and water to obtain a final reaction volume of 20 *μ*L.

Reactions were performed on the Abi Prism 7000 (Applied Biosystems, Foster City, CA, USA), with the following thermal cycler conditions: initial set-up of 10 min at 95°C, and 40 cycles of 15 s denaturation at 95°C and 1 min of annealing/extension at 60°C each. Samples were amplified in a 96-well plate. In each plate, endogenous control, samples and negative controls were analysed in triplicate. IL-25 and *β*-actin TaqMan gene expression primers were designed by Applied Biosystems and offered as inventoried assays (IL-25 ID: Mm00499822_m1; *β*-actin ID: Mm01205647_g1). Each assay contains 2 unlabelled primers and 1 6-FAM dye-labelled, TaqMan MGB probe. Primer concentration was optimized by a matrix of reactions testing a range of concentrations for each primer against different concentrations of the partner primer and also negative controls were included. Cycle threshold (*C*_t_) value was calculated for each sample, housekeeping and uninfected control. To normalize for differences in efficiency of sample extraction or cDNA synthesis we used *β*-actin as a housekeeping gene. To estimate the influence of infection on the gene expression levels we used a comparative quantification method (2^−ΔΔCT^). This method is based on the fact that the difference in threshold cycles (Δ*C*_t_) between the gene of interest and the housekeeping gene is proportional to the relative expression level of the gene of interest. The fold change in the target gene was normalized to *β*-actin and standardized to the expression at time 0 (uninfected animals) to generate a relative quantification of the expression levels.

### DNA extraction from stool samples

Total DNA was extracted from ~200 mg of fecal samples of individual mice by using the QIAamp Fast DNA Stool Mini Kit (51504, QIAGEN, Germany), according to the specifications from the manufacturer. The samples were immediately frozen and stored at −80°C until use in qPCR analysis of the gene 16S rRNA or massive sequencing.

### Determination of bacterial load in feces

To determine the total bacterial load present in fecal samples of animals subjected to dysbacteriosis prior to the induction of a secondary infection with *E. caproni* and compare it with animals in the presence of conventional secondary infection, qPCR of the 16S rRNA gene was performed. For this, the KAPA SYBR FAST qPCR kit (Sigma) was used. For each sample, a final PCR reaction volume of 20 *μ*L was prepared in duplicate, containing 2 *μ*L sample DNA, 10 *μ*L solution provided in the kit and 0.4 *μ*L forward and reverse primers (27F-PCRrtAGAGTTTGATCMTGGCTCAG; 338R-PCRrtTGCTGCCTCCCGTAGG AGT) at a final concentration of 0.2 mm. The reaction volume was completed with the addition of 7.2 *μ*L distilled water.

Prior to establishing the optimal conditions for qPCR, a PCR product of the 16S rRNA gene of *Enterococcus faecium* strain C68 was used to obtain a standard curve. This *E. faecium* 16S rRNA PCR was performed as follows. Briefly, a 25 *μ*L reaction solution was prepared containing 1 *μ*L 1 bacterial colony resuspended in phosphate-buffered saline, 2.5 *μ*L 10× Taq standard reaction buffer (New England BioLabs, Ipswich, MA, USA), 0.25 mm deoxynucleoside triphosphates, 2 0.5 U Taq DNA polymerases (New England BioLabs) and 0.2 mm primers. The volume was made up with water. The amplification conditions were an initial cycle of 5 min at 94°C and 35 cycles of denaturation for 30 s at 94°C, annealing for 30 s at 56°C and elongation for 30 s at 68°C, and a final extension cycle of 5 min at 68°C. Amplification was confirmed by electrophoresis by loading 4 *μ*L PCR reaction product on a 1.6% agarose gel. The remaining volume was purified using ExcelaPure™ 96-Well PCR Purification Plates (Edge Bio, San Jose, CA, USA).

The ENDMEMO program was used to determine the number of 16S rDNA molecules in the *E. faecium* C68 PCR product based on the 16S rRNA gene sequence and the concentration of the PCR product. A standard curve was obtained by making 5-fold dilutions of the PCR product. The qPCR cycling conditions were 94°C for 5 min and 45 cycles of 94°C for 30 s, 56°C for 30 s and 68°C for 30 s, and a final elongation cycle at 68°C for 30 s. By extrapolating the results with those obtained using the standard curve, the number of 16S rRNA genes was determined for each sample. The final number of 16S rRNA genes per g of fecal sample was calculated using the following formula:
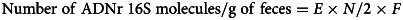
where *E* represents the volume of the buffer used for DNA dilution after extraction, *N* represents the number of 16S rDNA molecules obtained by qPCR, 2 represents the volume of DNA used for the qPCR reaction and *F* represents the weight (in g) from the fecal sediment from which the DNA was extracted.

### Bacterial 16S rRNA gene Illumina sequencing

Stool collection and DNA extraction was performed as described above. Afterwards, concentrations of DNA in stool samples were measured using a Qubit^®^ 2.0 Fluorometer (Life Technology, CA, USA) and normalized to 10 ng *μ*L^−^. Gut microbiota composition and diversity were determined by the V3–V4 variable region of the 16S rRNA gene sequencing. It was amplified by PCR using the Illumina protocol for the preparation of metagenomic sequencing libraries with the primers proposed by Klindworth *et al*. (2013) for detection of bacteria (forward: S-D-Bact-0564-a-S-15; reverse: S-D-Bact-0785-b-A-18; total coverage: 89%). Following the Illumina amplicon library protocol, DNA amplicon libraries were generated using a limited cycle PCR: initial denaturation at 95°C for 3 min, followed by 25 cycles of annealing (95°C for 30 s, 55°C for 30 s, 72°C for 30 s) and extension at 72°C for 5 min, using a KAPA HiFi HotStart ReadyMix (KK2602). Then, a Nextera XT index kit (Illumina, CA, USA) was used for the multiplexing step and a Bioanalyser DNA 1000 chip (Agilent Technologies, CA, USA) was used to check the PCR product quality. Libraries were sequenced using a 2× 300 pb paired-end run (MiSeq reagent kit v3) on a MiSeq-Illumina platform (FISABIO Sequencing Service, Valencia, Spain) according to the manufacturer's instructions (Illumina). For quality control, reagents employed for DNA extraction and PCR amplification were also sequenced. Taxonomy assignment was conducted using Silva138 database (Quast *et al*., 2013).

### Bioinformatics and statistical analysis

The evaluation of the quality of the sequences was carried out using the PRINSEQ-lite program (Schmieder and Edwards, 2011) that allows establishing a quality control and pre-processing of the genomic dataset quickly and easily from raw sequences in FAST or FASTQ format. A series of filters were established defining the minimum sequence length (min_length: 50); quality of the sequence cut (trim_qual_right: 20); sequence cut quality type (trim_qual_type: mean) and sequence cut quality window (trim_qual_window: 20). R1 and R2 from Illumina sequencing were joined using fastq-join from the ea-tools suite (Aronesty, 2011). Data were obtained using an *ad hoc* pipeline written in R Statistics environment (R Core Team, 2012) and data processing was performed using a QIIME pipeline (version 1.9.0) (Caporaso *et al*., 2010). Additional filters were applied: taxa with <3 reads in at least 20% of the samples, and taxa with <0.01% of the total reads in all samples were removed. In addition, the decontam package (Davis *et al*., 2018) in the R environment [Rizzo, 2019; RStudio Team (2020), RStudio: Integrated Development Environment for R, PBC, Boston, MA, USA] was used to find possible sequences related to contaminants. The clustered sequences were utilized to construct operational taxonomic unit tables with 97% identity and representative sequences were taxonomically classified at the taxonomic level of phylum, family and genus according to the data found in the Greengenes 16S rRNA gene database (version 13.8). Sequences that could not be classified to the domain level, or were classified as Cyanobacteria, Chloroplasts or Rhizobiales were removed from the dataset. All those sequences that could not be classified for a given taxonomic level were described at the previous taxonomic level with the prefix ‘Unclassified’. From these data, tables of relative abundance of taxa were generated for the different taxonomic levels (phylum, family and genus). Subsequently, *α* diversity indices (Chao1 and Shannon, species richness estimates and diversity index, respectively) and *β* diversity indices based on the Bray–Curtis distance (nonphylogenetic) between groups at the genus level were studied and permutational multivariate analysis of variance was used to test significance. Calypso software version 8.84 (http://cgenome.net/wiki/index.php/Calypso/) was used with total sum normalization for the statistical analysis, and also, cumulative sum scaling normalization for multivariate test (redundancy discriminant analysis – RDA) as well as principal coordinate analysis (PCoA).

Moreover, to identify taxa with differentiating abundance in the different environments the linear discriminant analysis (LDA) effect size (LEfSe) algorithm was used with the online interface Galaxy (http://huttenhower.sph.harvard.edu/lefse) (Segata *et al*., 2011). LEfSe couples robust tests for measuring statistical significance (Kruskal–Wallis test) with quantitative tests for biological consistency (Wilcoxon-rank-sum test). The differentially abundant and biologically relevant features are ranked by effect size after undergoing LDA. An effect size threshold between 2 and 3 (on a log_10_ scale) was used for all biomarkers discussed in this study.

Additionally, Student's *t*-test was used to compare worm recoveries and IL-25 expression. One-way analysis of variance with Bonferroni test as post-hoc analysis was used to compare expression levels of IL-25 and bacterial loads in feces. The *P* < 0.05 value was considered as significant. Prior to analyses, data were log-transformed to achieve normality and verified by using the Anderson–Darling test.

## Results

### Experimental infection outcomes

**Fig. 1.**
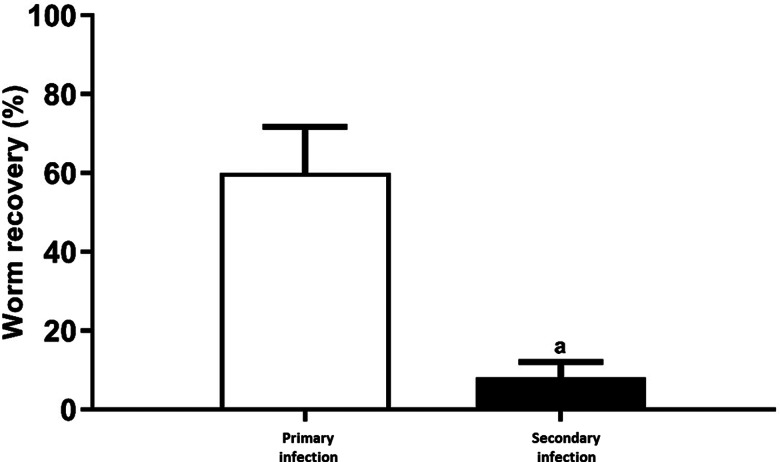
Worm recovery expressed as percentage in primarily and secondarily infected mice with metacercariae of *Echinostoma caproni*. a: significant differences between infected and secondarily infected mice (*P* < 0.001).

The results obtained herein show that ICR mice develop partial resistance against homologous challenge infection with *E. caproni* on the basis of worm recovery (Fig. 1). All the mice exposed to 50 metacercariae of *E. caproni* were infected and were positive to egg detection and necropsy at 10 wppi. Worm recovery was significantly higher in animals primarily infected than those exposed to metacercariae after pzq treatment (Fig. 1). Worm recovery in primarily infected mice ranged from 19 to 42 worms per mouse (27.1 ± 8.7), whereas a reduction of 86.8% was observed in the secondarily infected mice with 1–9 worms per mouse (3.6 ± 3.8). Application of the Student's *t*-test to the worm recovery showed that the values of worm recovery were significantly lower in secondary animals than in infected mice (*P* < 0.001).

### Effect of primary infection, treatment with pzq and secondary infection in the production of IL-25

**Fig. 2.**
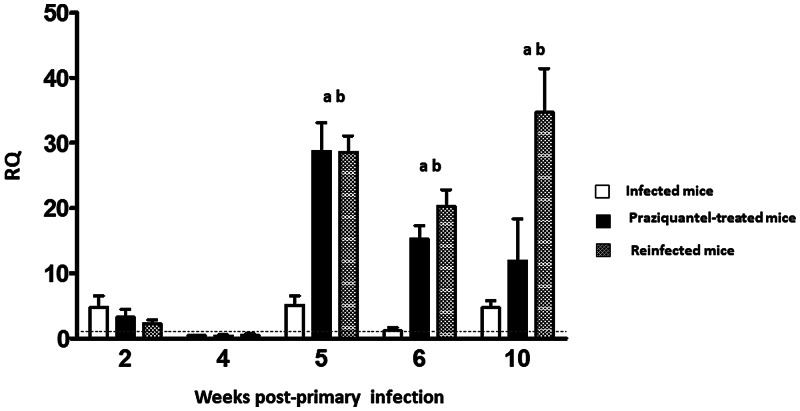
Expression of IL-25 mRNA in the intestinal tissue of ICR mice infected, pzq-treated and reinfected with *E. caproni*. The relative quantities (RQ) of cytokine genes are shown after normalization with *β*-actin and standardization of the relative amount against day 0 sample. The vertical bars represent the standard deviation. a: significant differences with respect to negative controls; b: significant differences between groups at each week of the study (*P* < 0.05).

Our results confirm that primary *E. caproni* infection in mice does not induce significant changes in the production of IL-25. No significant changes in IL-25 production were observed in any of the 3 groups of mice used to study IL-25 production. In fact, no change in IL-25 expression was observed during the entire experiment in that group of mice that were only subjected to a primary infection (Fig. 2). However, significant increases in IL-25 expression were detected in the pzq-treated and reinfected groups of mice as a consequence of treatment with pzq and healing of the primary infection (*P* < 0.05). IL-25 expression levels remained high in both groups of mice until the end of the experiment at 10 wppi (Fig. 2).

### Effect of resident microbiota on the production of IL-25 and resistance to infection

**Fig. 3.**
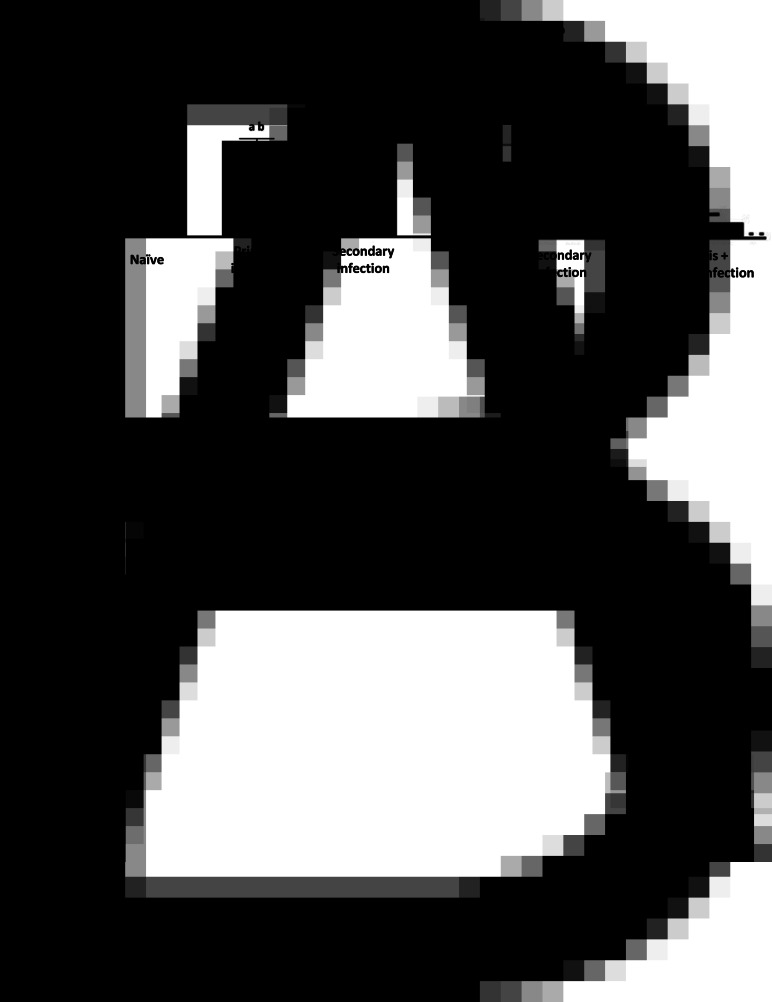
Quantitative changes in the composition of the resident microbiota in the intestine of mice subjected to primary and secondary infections with *E. caproni* and expression of IL-25 in the presence or absence of dysbiosis. (A) Quantitative evaluation of the resident microbiota in the intestine of mice in the presence of primary and secondary infections with *E. caproni* analysed by qPCR of the 16S rRNA gene of *Enterococcus faecium* C68 strain expressed as 16S rRNA genes per mg of fecal sample. (B) Relative expression of IL-25 mRNA in the intestinal tissue of secondary infected mice with or without previous antibiotic treatment to induce dysbiosis. The RQs of cytokine genes are shown after normalization with *β*-actin and relative amount of standardization against naïve mice. The vertical bars represent the standard deviation. a: significant differences with respect to naïve mice; b: significant differences between groups (*P* < 0.05).

To analyse the influence of intestinal resident microbiota in the non-specific upregulation of IL-25 after cure of primary infection, the changes in the bacterial load as a consequence of the infection was initially analysed by reverse transcription qPCR. The results obtained showed that exposure to primary *E. caproni* infection induced a significant quantitative reduction of bacterial load. Nevertheless, after pzq treatment and cure of the primary infection the bacterial charge was suddenly recovered (Fig. 3A).

To further confirm the involvement of intestinal microbiota in the regulation of IL-25 expression, we treated a group of mice at 4 wppi with a cocktail of broad-spectrum antibiotics to induce dysbacteriosis and challenged at 6 wppi. All mice were sacrificed at 10 wppi. Analysis by qPCR showed that antibiotic-treated mice did not produce IL-25 in response to reinfection with *E. caproni*. In contrast, non-treated mice responded with elevated levels of IL-25 gene expression to secondary exposure to metacercariae of *E. caproni* (Fig. 3B).

### Fecal bacterial profile

After filtration and chimaera removal, our dataset contained 6 426 398 reads (min–max 106 229–227 068). The resulted phyloseq object consisted of a total of 43 different genera catalogued in the Silva138 database (Quast *et al*., 2013). Furthermore, the retrieved genera were distributed among 9 phyla and 36 families.

**Fig. 4.**
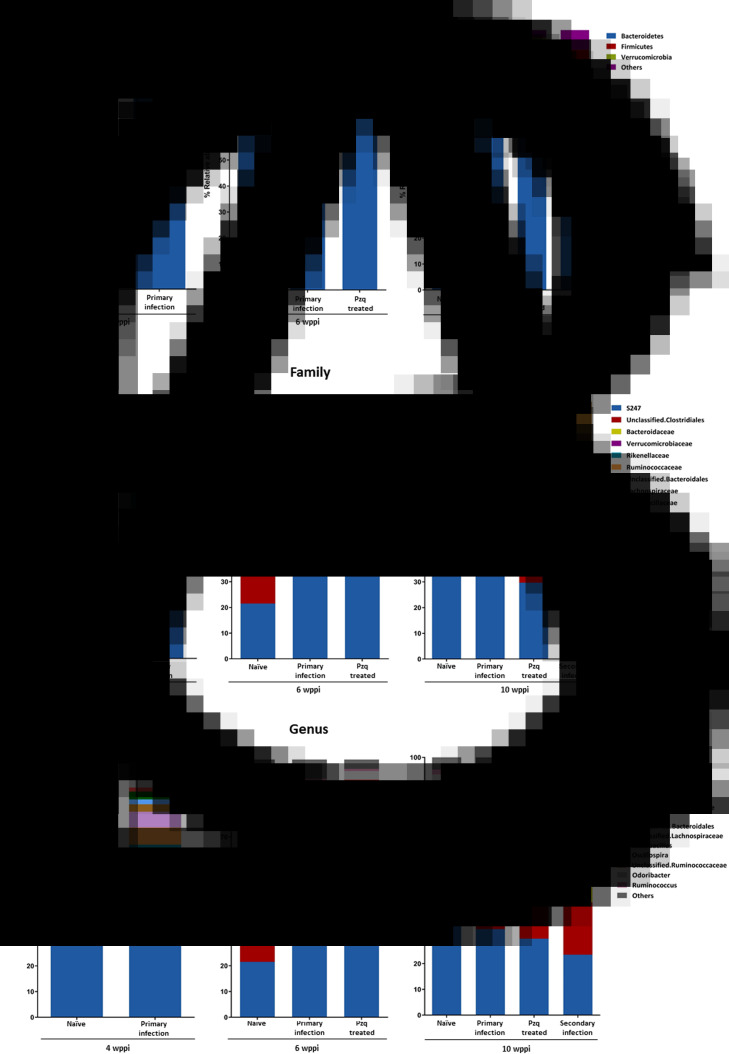
Analysis of the relative abundance of the main phyla, families and genera detected in fecal samples from mice under different experimental conditions at 4, 6 and 10 wppi. (A–C) Relative abundance at the phylum, family and genera levels (expressed as a percentage at 4, 6 and 10 weeks from the beginning of the experiment) detected in naïve, primarily infected with *E. caproni*, pzq-treated and secondarily infected mice.

For brief characterization, the most abundant phyla, families and genera are presented in Fig. 4A–C and further information in relation to the relative abundance of bacteria in each group of animals can be found in Supplementary Tables S1–S3.

Bacteroidetes followed by Firmicutes were the most abundant phyla in all the experimental groups. At the family level, S247, Unclassified Clostridiales and Unclassified Bacteroidaceae were the most represented in all samples. Likewise, all experimental groups shared a core microbiota composed of the genera *Unclassified S247*, *Unclassified Clostridiales*, *Bacteroides*, *Akkermansia*, *Unclassified Rikenellaceae*, *Prevotella*, *Unclassified Bacteroidales*, *Unclassified Lachnospiraceae*, *Lactobacillus*, *Oscillospira*, *Unclassified Ruminococcaceae*, *Odoribacter* and *Ruminococcus* (Fig. 4C). In contrast, members of the genera *Turicibacter* and *Bifidobacterium* were exclusively detected in pzq-treated mice in each week of the experiment. The genera *Unclassified S247* and *Unclassified Clostridiales* were the most prevalent in all samples and in all experimental groups.

### Impact of primary infection, pzq treatment and secondary infection on gut microbiota profile

All the experimental procedures induced changes in the gut microbiota of mice. The composition of the intestinal microbiota analysed from feces obtained from mice infected with *E. caproni* at the different experimental times showed statistically significant differences at the phylum, family and genera levels (Fig. 4A–C). More detailed data regarding the percentage proportion of bacterial phyla and at each fecal sample are presented in Supplementary Tables S1–S3.

**Fig. 5.**
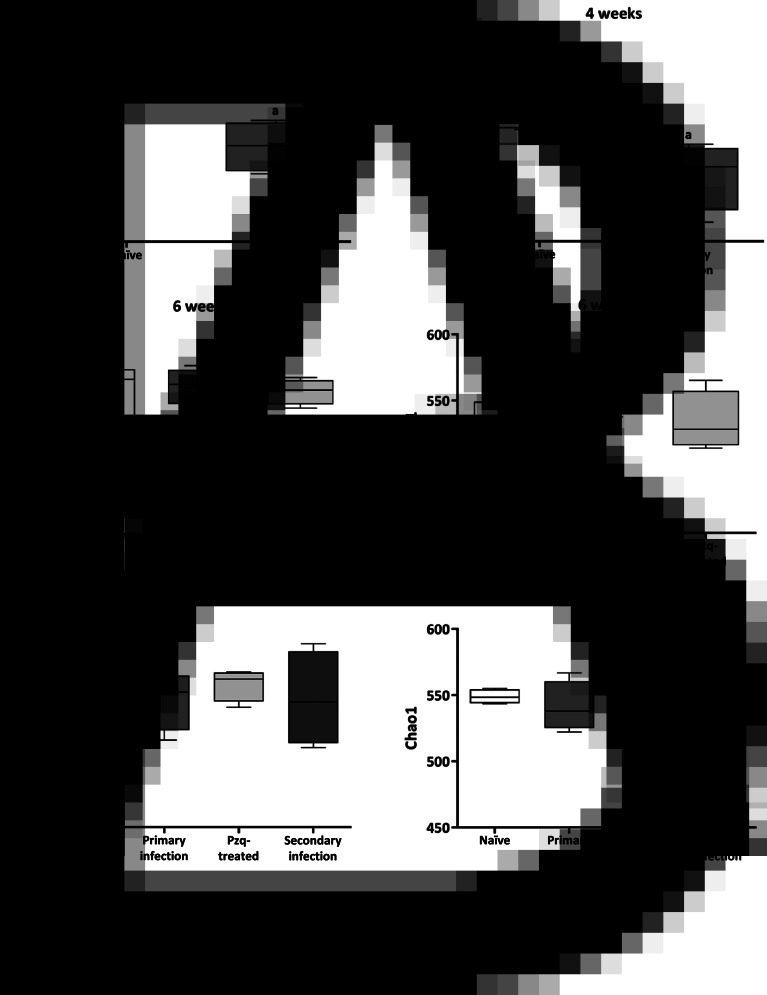
Analysis of *α*-diversity and microbial richness in fecal samples from mice in the presence and/or absence of infection with *E. caproni* at different experimental times. The *α*-diversity was measured from the microbiota detected in fecal samples using the Shannon index (A) and the richness using the Chao1 index (B) at 4, 6 and 10 weeks from the beginning of the experiment in naïve, primarily infected with *E. caproni*, pzq-treated and secondarily infected mice. a: significant differences between naïve and infected mice with *P* < 0.05.

Differences in the *α*-diversity in the 4 experimental groups were determined by using the Shannon index. Mice with primary infection showed a moderately low diversity at 4 wppi compared to naïve mice in the same experimental week (*P* < 0.05) (Fig. 5A). Naïve, infected, pzq-treated and secondarily infected mice showed similar *α*-diversity values at both 6 and 10 weeks from the beginning of the experiment and no significant differences were observed in *α*-diversity over the course of the experiment. On the other hand, the richness of the microbiota of infected mice at 4 wppi, evaluated by the Chao1 index (Fig. 5B), was quite low compared to naïve animals at the same experimental time (*P* < 0.05). In contrast, no significant differences were observed in neither intra- nor inter-groups or within during different weeks of study.

However, in mice exposed to reinfection, a greater variability in diversity in samples within the group was observed. No significant differences were observed in *α*-diversity over the course of the experiment after primary infection between groups.

**Fig. 6.**
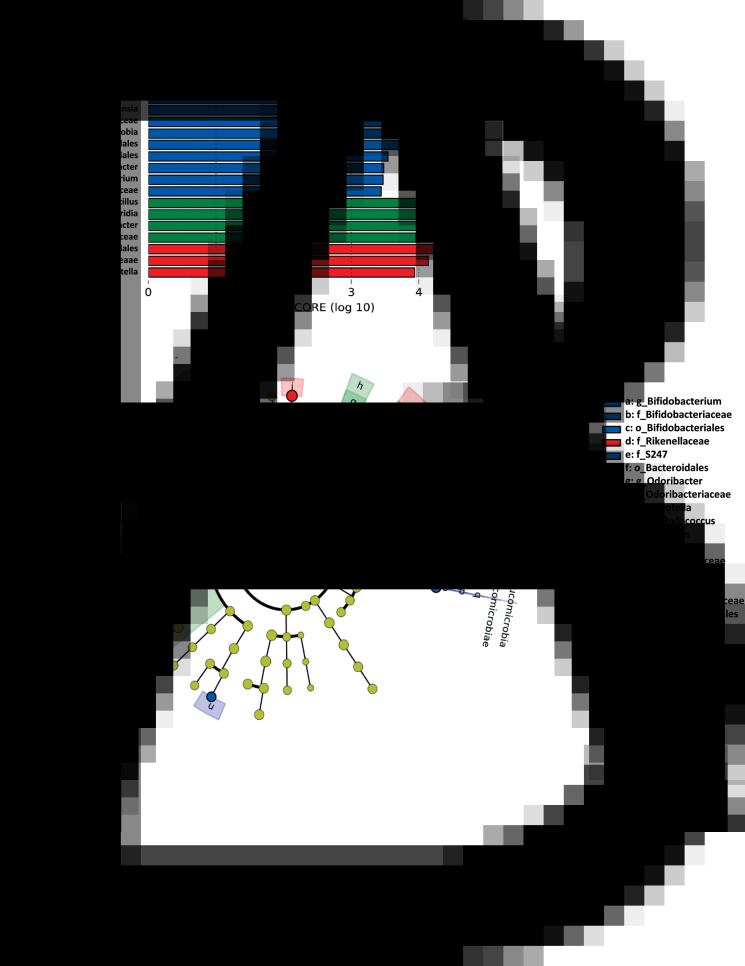
Analysis of the bacterial taxa showing significant differences in the relative abundance of the microbial profiles obtained from fecal samples of naïve, primarily infected with *E. caproni*, pzq-treated and secondarily infected mice, according to LEfSe. (A) Significant differences obtained in the relative abundance of the main microbial taxa detected in feces of infected mice with *E. caproni* (red), pzq-treated (blue) and secondarily infected (green) with an LDA score of >2. (B) Cladogram representation of the significant differences obtained from the LEfSe analysis in the relative microbial abundance detected in the feces of infected (red), treated (blue) and secondarily infected (green) mice.

LEfSe) showed that high abundance of the order Bacteroidales (LDA 4.56, *P* < 0.05), family Rikenellaceae (LDA 4.13, *P* < 0.05) and the genus *Prevotella* (LDA 3.93, *P* < 0.05) were characteristics of infected mice (Fig. 6). However, bacteria belonging to the phylum Verrucomicrobia (LDA 4.11, *P* < 0.05), order Verrucomicrobiales, Bacillales and Bifidobacteriales (LDA 4.41, 3.75 and 3.54, respectively, *P* < 0.05) and families S247, Verrucomicrobiaceae, Lachnospiraceae and Bifidobacteriaceae (LDA 4.81, 4.11, 4.18 and 3.44, respectively, *P* < 0.05) were distinctive of the pzq-treated mice group. In the same way, genera *Staphylococcus*, *Akkermansia*, *Turicibacter* and *Bifidobacterium* (LDA 4.22, 4.12, 3.48 and 3.48, respectively, *P* < 0.05) were prevalent in this group of mice. The family Odoribacteriaceae (LDA 3.95 *P* < 0.05), the class Clostridia (LDA 4.31 *P* < 0.05) and genera *Lactobacillus* and *Odoribacter* (LDA 4.52 and 3.95, respectively, *P* < 0.05) were found to be significantly enriched in secondarily infected mice compared to the remaining groups of mice.

Exposure to primary *E. caproni* infection induced significant changes in the resident microbiota of infected mice that can be observed at different wppi. The composition of the intestinal microbiota analysed from feces obtained from infected mice with *E. caproni* at the different experimental time showed statistically significant differences at the phylum and family levels (Fig. 4A and B). However, the gut microbiota composition was rather homogeneous over the course of primary infection and no significant changes were detected until 10 wppi in this group. Exposure to primary *E. caproni* infection caused a significant increase in the relative abundance of the members of the phylum Bacteroidetes (71.72 *vs* 61.61%) concomitantly with a reduction of the phylum Firmicutes (22.62 *vs* 35.21%) at 4 wppi with respect to naïve mice (*P* < 0.05) (Fig. 4A).

The diversity detected in the microbiota at the family level was evident in samples collected under all experimental conditions. The 9 most abundant bacterial families in the microbiota of all experimental groups analysed are presented in Fig. 3B. More detailed data regarding the percentage proportions of bacterial families at each fecal sample are presented in Supplementary Table S2. The bacterial families S247, Unclassified Clostridiales and Bacteroidaceae were the most abundant contributing together around 49.71–67.78% of the global microbiota composition in all experimental group as mean value (Fig. 4B). Moreover, at the family level, a reduction in the relative abundance of Unclassified Clostridiales (11.75 *vs* 22.38%) was observed, concurrently with an increase in the abundance of Unclassified Bacteroidales (6.17 *vs* 1.95%) in infected mice at 4 wppi with respect to naïve mice at the same experimental time (*P* < 0.05) (Fig. 4B). At 6 wppi, a significant increase in the S247 family (33.64 *vs* 21.51%) was observed in infected mice as compared to naïve mice (*P* < 0.05). Likewise, in primarily infected mice, a significant increase in the relative abundance of the genera *Bacteroides*, *Prevotella* and *Unclassified Bacteroidales* (13.43 *vs* 7.47%, 6.64 *vs* 3.8% and 6.17 *vs* 1.95%, respectively) was detected, as well as a decrease in *Unclassified Clostridiales* (11.75 *vs* 25.19%) with respect to naïve mice at 4 wppi (*P* < 0.05). On the other hand, at 6 wppi levels of *Unclassified S247* (33.66 *vs* 21.51%) were significantly increased in relation to naïve mice (*P* < 0.05). In addition, the significant increase in the genus *Unclassified Bacteroidales* (8.05 *vs* 3.05%) observed at 4 wppi in infected mice compared to naïve mice appeared again at 10 weeks of the experiment (*P* < 0.05). However, the gut microbiota composition was rather homogeneous over the course of primary infection.

An enrichment in the relative abundance of the phylum Verrucomicrobia was observed in pzq-treated mice at both 6 wppi (3.04 *vs* 0.08%) (*P* < 0.001) and 10 wppi (2.54 *vs* 0.44%) (*P* < 0.05) and compared to age-matched naïve mice. In addition, at the family level, a significant increase in the relative abundance of S247 (44.60 *vs* 21.51%) was detected at 6 wppi (*P* < 0.001) as well as that of the Verrucomicrobiaceae family both at 6 and 10 wppi (3.04 *vs* 0.08% and 2.54 *vs* 0.43%, respectively) from the start of the experiment with respect to naïve mice (*P* < 0.05). However, these animals showed a significant reduction in the relative abundance of the families Bacteroidaceae, Rikenellaceae and Unclassified Bacteroidales compared to what was observed in naïve mice at 6 wppi (6.03 *vs* 12.54%, 8.24 *vs* 13.5% and 0.26 *vs* 2.65%, respectively) (*P* < 0.05), and this reduction was constant until the end of the experiment in the Unclassified Bacteroidales family (0.27 *vs* 3.05%) (*P* < 0.05). These changes in the microbiota composition of pzq-treated mice were also observed at the genus level where a significant increase in the relative abundance of the genera *Unclassified S247* and *Akkermansia* (44.60 *vs* 21.51% and 3.04 *vs* 0.08%, respectively) was found and at the same time *Unclassified Rikenellaceae* (8.24 *vs* 13.01%) decreased with respect to naïve mice at 6 wppi of the experiment (*P* < 0.05). The increase in the genus *Akkermansia* (2.54 *vs* 0.01%) was maintained at 10 wppi of the experiment, at which time there was a decrease in the genus *Unclassified Bacteroidales* (0.27 *vs* 3.05%) in relation to what was detected in naïve mice (*P* < 0.05).

### Impact of pzq treatment

**Fig. 7.**
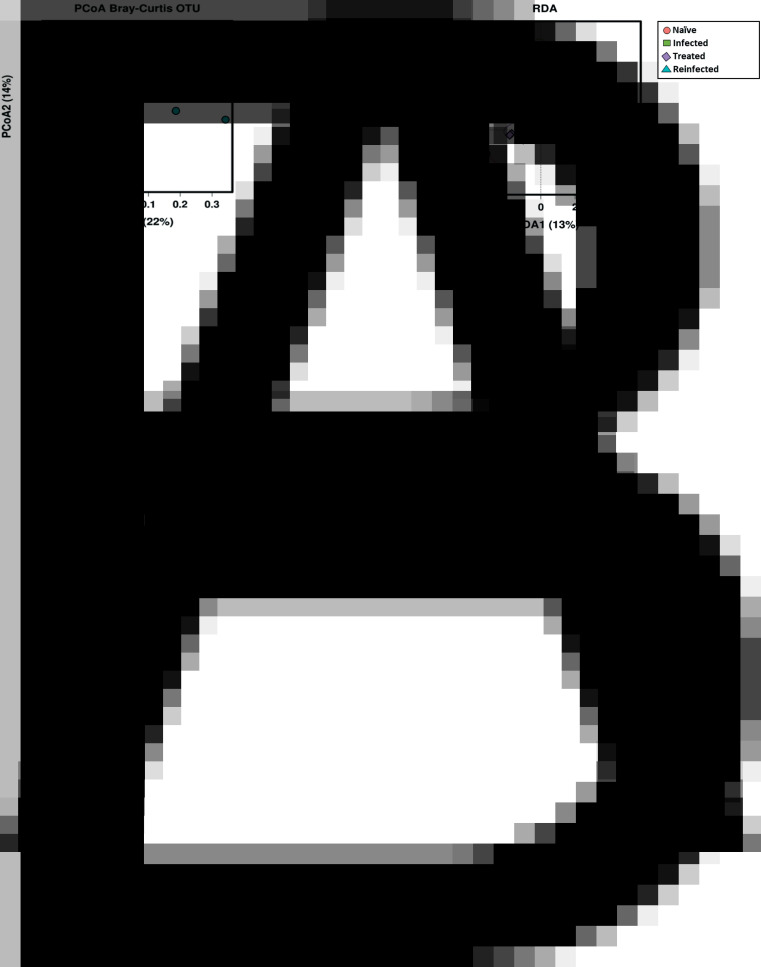
PCoA Bray–Curtis (A) and RDA. Microbial profile obtained from fecal samples from naïve, primarily infected with *E. caproni*, pzq-treated and secondarily infected mice at 10 weeks of the experiment.

PCoA of the samples corresponding to pzq-treated mice were clearly separated and showed greater variability than that observed in the infected mice (Fig. 7A). In the RDA, no significant interaction was observed between the microbiota of primary infected and pzq-treated mice (Fig. 7B).

A comparison of the relative abundance of bacterial taxa after healing of the primary infection with the data observed in infected mice showed a significant increase of the *Unclassified Lachnospiraceae* (4.37 *vs* 2.48%) and a decrease of *Lactobacillus* (1.83 *vs* 5.46%) and *Unclassified Bacteroidales* (0.27 *vs* 8.05%) at 10 wppi (*P* < 0.05).

Probably, the most striking feature observed in the pzq-treated mice was the marked increase in the relative abundance of Verrucomicrobia exclusively represented by the members of genus *Akkermansia* (3.04 *vs* 0.02%) and this abundance was constant in the pzq-treated mice until the end of the experiment (2.54% *vs* non-detected) (Fig. 3C). A similar pattern followed the members of the genera *Bifidobacterium* (0.13% *vs* non-detected) and Turicibacter (0.5% *vs* non-detected), although the increase after treatment was less pronounced (Supplementary Table S3).

### Impact of secondary infection

The microbial profile detected by the PCoA in the samples corresponding to secondarily infected mice clustered separately from the rest of the other experimental groups and showed greater variability than that observed in infected mice (Fig. 7A). In the RDA, no significant interaction was observed between the microbiota of infected and secondarily infected mice (Fig. 7B).

Secondary infection of the pzq-treated animals was characterized by the complete abrogation of the members of the phylum Verrucomicrobia. Moreover, significant increase of the relative abundances of the families Unclassified Bacteroidales (4.85 *vs* 0.27%) and Lactobacillaceae (4.52 *vs* 1.83%) (*P* < 0.05) was detected at 10 wppi. At the genus level, reinfection was characterized by a significant reduction in the relative abundance of *Unclassified Lachnospiraceae* (1.62 *vs* 4.37%) (*P* < 0.05) and the complete disappearance of the genera *Akkermansia*, *Bifidobacterium* and *Turicibacter.* Concomitantly, a significant increase of the members of *Lactobacillus* (8.04 *vs* 1.83%) was observed (*P* < 0.05).

These facts entailed a return to a similar profile to that observed in primary infection, characterized by predominance of the phyla Bacteroidetes and Firmicutes and the complete abrogation of the members of the phylum Verrucomicrobia. The most represented families were S247, Unclassified Clostridiales and Bacteroidaceae. Only a decrease in the relative abundance of Unclassified Bacteroidales (4.85 *vs* 8.05%) (*P* < 0.05) was observed at 10 wppi. Moreover, 32 genera out of a total of 33 were common in both groups of animals.

## Discussion

There is growing evidence supporting the cross-talking among parasites, hosts and resident microbiota plays a critical role in the outcome of intestinal helminth infections by affecting mechanisms of parasite establishment or modulation of the host immune responses (Li *et al*., 2016; El-Ashram *et al*., 2017; Cortés *et al*., 2020). In this context, the experimental model of *E. caproni* mouse offers the possibility to study the role of microbiota in processes such as susceptibility and resistance to infection (primary and secondary infections, respectively) and cure of infection.

Susceptibility of mice to *E. caproni* infection depends on the ability of the host to respond to IL-25 production (Muñoz-Antoli *et al*., 2016*a*; Álvarez-Izquierdo *et al*., 2020*a*). Primary infections become chronic and characterized by elevated levels of inflammation and tissue damage in relation to the lack of IL-25 production (Muñoz-Antoli *et al*., 2007). Pharmacological curation of the primary infection abruptly elevated the levels of IL-25 conferring resistance to homologous secondary infection (Muñoz-Antoli *et al*., 2016*a*, 2016*b*). Considering that several studies support that IL-25 production is mediated by signals derived from gut microbiota (Sawa *et al*., 2011; Donaldson *et al*., 2015; Watanabe *et al*., 2017), we analyse the changes in the resident microbiota induced by a primary *E. caproni* infection and curation and a secondary infection.

Treatment of mice with a cocktail of antibiotics abrogated the IL-25 response after the curation of the primary *E. caproni* infection concomitantly with a decrease in bacterial abundance in feces and susceptibility to challenge infection at 2 wppi. This indicates that resident microbiota may play a pivotal role in the expression of IL-25 and, consequently, in the resistance to challenge infections. This is consistent with previous studies showing that IL-25 production is dependent on microbial-derived signals (Sawa *et al*., 2011; Donaldson *et al*., 2015; Watanabe *et al*., 2017).

Primary *E. caproni* infection in mice induced a significant reduction of *α*-diversity, which is consistent with the fact that primary infection causes chronic infections with elevated levels of inflammation and tissue damage (Toledo *et al*., 2006; Muñoz-Antoli *et al*., 2007). *α*-Diversity is commonly used as a measurement to determine the health status of the microbiota. High values of *α*-diversity are associated with mature, homogeneous, stable and healthy intestinal microbial environment (Mosca *et al*., 2016; Menni *et al*., 2017). Consequently, it has been proposed that the direct or immune-mediated ability of gastrointestinal helminths to restore intestinal homoeostasis by promoting increases in microbial richness and uniformity may represent a mechanism by which parasites exert therapeutic properties in individuals with chronic inflammatory disorders (Giacomin *et al*., 2015, 2016; Zaiss *et al*., 2015; Jenkins *et al*., 2017).

This reduction was confirmed by the Chao1 index. The richness of the intestinal microbiota of mice in the presence of primary infection at 4 wppi evaluated by the Chao1 index was lower than that observed in control animals at the same time point. Likewise, this index increased in all experimental groups both at 6 and 10 wppi and no significant differences were observed between groups or within the same group at different weeks of study. This fact suggests that the individual microbiota of mice with primary and secondary infections at 6 and 10 wppi differentially responded to the alteration of intestinal homoeostasis observed in the early phases of primary infection. Previous studies have detected a significant increase in microbial richness in the gut microbiota of laboratory rodents experimentally infected with *Trichuris suis* and *Hymenolepis diminuta* (Holm *et al*., 2015; Kreisinger *et al*., 2015), as well as in fecal samples from humans infected with various intestinal helminth species (Jenkins *et al*., 2017). These data suggest that a higher richness could represent a common characteristic given in the intestinal microbiota of mammals parasitized by helminths, regardless of the species of parasites and the location in the gastrointestinal tract. However, in addition to the state of infection (acute or chronic), together with the characteristics of the parasite species in general, it is likely that the parasite load also affects the changes induced by intestinal helminths in the intestinal microbial richness (Jenkins *et al*., 2017).

Moreover, analysis of *β*-diversity supports the relevant role of helminth infection in the composition of resident microbiota. The taxonomic profile of microbial communities, using PCoA and RDA, revealed strong associations between intestinal composition and infection stage (primary or secondary infection), thus providing further evidence of the modulating function of the microbiota (directly or immune-mediated) in infections caused by intestinal helminths (Jenkins *et al*., 2018*a*). Moreover, LEfSe analysis showed that all of the experimental procedures markedly affected the richness of microbial communities with the major changes observed in the populations of Lachnospiraceae, Rikenellaceae, Unclassified Clostridiales, Unclassified Bacteroidales, *Lactobacillus*, *Akkermansia*, *Prevotella* and *Odoribacter*.

The overall observation of the microbiota profile in each of the 4 experimental groups shows that Bacteroidetes and Firmicutes were the most represented phyla in all the groups. However, each experimental procedure elicited significant changes that may contribute to explain the susceptibility or resistance to *E. caproni* infection.

Primary infection with *E. caproni* induced a significant increase in the relative abundance of Bacteroidetes to the detriment of the Firmicutes, affecting the Bacteroidetes/Firmicutes ratio (1.75 in naïve *vs* 3.17 in primarily infected mice). Growth of Bacteroidetes is mainly related to an increase of Unclassified Bacteroidales. It has been suggested that intestinal helminths, such as *Trichuris* spp., enhance to expansion of Bacteroidetes which may contribute to the restoration of the intestinal tissue damages by the helminth infection (Giacomin *et al*., 2015; Myhill *et al*., 2018).

Members of Bacteroidetes are involved in the degradation of carbohydrates and proteins, suggesting that primary *E. caproni* infection induces an increase of the metabolism of this type of molecules as previously described for other helminths. Increase of Bacteroidetes/Firmicutes ratio has been commonly associated with the growth of beneficial bacteria enhancing anti-inflammatory responses and may induce several metabolic changes in the intestine (Myhill *et al*., 2018). The increase of Bacteroidetes induced by primary *E. caproni* infections is consistent with the metabolic changes previously observed. Primary infection of mice with *E. caproni* affects the carnitine biosynthetic pathway, which induces an increase of *β*-oxidation of fatty acids as an alternative source of energy (Cortés *et al*., 2015). Bacteroidetes has the ability to use a large range of substrates to degrade carbohydrates as a source of energy (Thomas *et al*., 2011). In this context, the release of fatty acids as a consequence of the anaerobic degradation of carbohydrates by members of Bacteroidetes may facilitate the procurement of additional energy to the host.

Moreover, the decrease of Firmicutes may contribute to the accumulation of short-chain fatty acids and the inflammation observed in primary *E. caproni* infections. Several studies have demonstrated that the members of Firmicutes are able to degrade pro-inflammatory factors, reduce cellular infiltration and reactive oxygen species levels, ameliorating the intestinal inflammation (Mannick and Udall, 1996; von Schillde *et al*., 2012; Hörmannsperger *et al*., 2013; Yin *et al*., 2018). Thus, reduction of Firmicutes may favour the pathology induced by primary *E. caproni* infections due to an increase of inflammatory responses, oxidative stress and accumulation of short-chain fatty acids which are relevant factors causing pathology (Cortés *et al*., 2015).

Increased populations of Clostridia were detected in the fecal microbiota of mice during primary infection with *E. caproni* compared to subjects treated with pzq or subjected to secondary infection. Several strains of Clostridia have been identified as main actors in the maintenance of intestinal homoeostasis, due to their role in the protection of the intestine against the colonization of pathogens, as a mediator of the development of the host's immune system and as a modulator of the immunological tolerance (Lopetuso *et al*., 2013; Jenkins *et al*., 2018*b*).

Pharmacological curation of primary *E. caproni* infection rapidly activates mechanisms for wound healing, including the development of a Th2 phenotype with elevated levels of IL-25 and alternative activation of macrophages and the transition from an inflammatory to anti-inflammatory milieu (Cortés *et al*., 2015; Muñoz-Antoli *et al*., 2016*a*, 2016*b*). Strikingly, this process is associated with a marked increase of beneficial microbes such as those of the Verrucomicrobia phylum (Verrucomicrobiaceae) together with a depletion of native bacteria including Bacteroidaceae or Unclassified Bacteroidales. Increased abundance of Verrucomicrobia has been associated with the development of Th2 phenotype in response to *Trichinella spiralis* infections (Liu *et al*., 2019). It has been shown that Verrucomicrobia cause a decrease in inflammation and enhanced glucose metabolism of the host (Fujio-Vejar *et al*., 2017; Plovier and Cani, 2017; Liu *et al*., 2019; Fujisaka *et al*., 2020; Ray *et al*., 2021). Shift from the Th1 to Th2 phenotype in *E. caproni* infections after pharmacological curation is characterized for an increased aerobic use of glucose (Cortés *et al*., 2015; Muñoz-Antoli *et al*., 2016*a*, 2016*b*). In addition, it has been shown that *Akkermansia muciniphila* adheres to the intestinal epithelium and strengthens the integrity of enterocytes *in vitro* assays (Justus *et al*., 2015). Therefore, it is plausible that high levels of *A. muciniphila* may play a potential protective role against the alteration of the barrier function of the epithelium after infection (Jenkins *et al*., 2018*a*).

Although it is difficult to establish a relationship between changes in the microbiota with the abrupt production of Th2 type cytokines (IL-4, IL-13) and IL-25 and resistance, the elevation of IL-25 levels occurred concomitantly with an increase of the Verrucomicrobia belonging to the genus *Akkermansia* and a dramatic decline in the Unclassified Bacteroidales. Interestingly, increase in the members of the genus *Akkermansia* has been associated with increased expression of Toll-like receptors and inflammatory genes (Ray *et al*., 2021). This suggests that the elevated levels of *Akkermansia* in our study are related to the inflammatory events induced by primary *E. caproni* infection. In contrast, reduced abundance of members of Bacteroidetes phylum has been associated with a decay in the inflammatory cytokines (Han *et al*., 2020). Therefore, the reduction of Unclassified Bacteroidales after pharmacological curation of the primary *E. caproni* infection could contribute to the increase of Th2 cytokines mediated by alarmins such as IL-25.

Several bacterial taxa, such as the family Lachnospiraceae or bacteria belonging to the genus *Turicibacter* (Firmicutes) were increased after pzq treatment. *Turicibacter* spp. were exclusively found in mice treated with pzq. Unfortunately, there is scanty information about the relationships between this group of bacteria and gastrointestinal helminth infections. However, in immunocompromised mice, a link between *Turicibacter* and host immune dysfunction has been described (Presley *et al*., 2010; Dimitriu *et al*., 2013). For example, *Turicibacter* spp. are abundant in the gut microbiota of wild-type mice, but are completely absent in the gut of mice with defective immune responses (CD45-) and mice lacking an adaptive immune system (RAG-) (Dimitriu *et al*., 2013). Consequently, we suggest that the absence of *Turicibacter* observed in mice infected with *E. caproni* could be due to alterations in the immune functions of the mucosa during infection. However, it is not clear whether the absence of bacteria belonging to the genus *Turicibacter* could have an impact on the outcome of *E. caproni* infection.

Secondary *E. caproni* infection completely depleted the phylum Verrucomicrobia for the benefit of a marked increase of Unclassified Bacteroidales. Among Firmicutes, members of the genus *Lactobacillus* showed a marked increase. A relationship between lactobacilli and parasitic helminths has been observed in experimental infections of murine models with intestinal nematodes (Walk *et al*., 2010; Rausch *et al*., 2013; Reynolds *et al*., 2014; Fricke *et al*., 2015; Kreisinger *et al*., 2015; Su *et al*., 2018). Reynolds *et al*. (2014) reported a marked increase in the populations of the Lactobacillaceae family after infection of C57BL/6 mice with *Heligmosomoides polygyrus*. In turn, the administration of *Lactobacillus* species prior to infection with the parasite resulted in a significant increase of parasite load, which led the authors to hypothesize the existence of a mutualistic relationship mediated by the immune system between selected bacteria and helminths (Reynolds *et al*., 2014). Strikingly, our results suggest that lactobacilli are affected by the inflammatory responses caused by the infection and, therefore, the elevated levels of *Lactobacillus* after secondary infections of mice may be a modulation of the local microbiota in response to infection.

## Conclusions

Herein, we have shown that primary *E. caproni* infection, healing and challenge infection have a great impact on the composition of the resident microbiota, which may be related to the outcome of the infection. Interestingly, both primary and challenge infection depleted the presence of members of Verrucomicrobia that only appeared as a consequence of the curation of the primary infection, concomitantly with the upregulation of IL-25 and the subsequent resistance to infection. Although further studies are required to exactly determine the role of intestinal microbiota in the course of helminth infections, our results suggest a close relationship between the changes in microbiota and the outcome of helminth infection.

## Data Availability

The datasets generated and/or analyzed during the current study are available from the corresponding author on reasonable request.

## Supplementary material

For supplementary material accompanying this paper visit https://doi.org/10.1017/S0031182022001366. click here to view supplementary material
